# Carotenoids and Some Other Pigments from Fungi and Yeasts †

**DOI:** 10.3390/metabo11020092

**Published:** 2021-02-06

**Authors:** Alexander Rapoport, Irina Guzhova, Lorenzo Bernetti, Pietro Buzzini, Marek Kieliszek, Anna Maria Kot

**Affiliations:** 1 Laboratory of Cell Biology, Institute of Microbiology and Biotechnology, University of Latvia, Jelgavas Str. 1-537, LV-1004 Riga, Latvia; 2 Laboratory of Cell Protective Mechanisms, Institute of Cytology, Russian Academy of Sciences, Tikhoretsky Avenue 4, 194064 Saint Petersburg, Russia; irina.guzh@gmail.com; 3 Department of Agricultural, Food and Environmental Sciences and Industrial Yeasts Collection DBVPG, University of Perugia, Borgo XX Giugno 74, 06121 Perugia, Italy; lorenzo.bernetti@gmail.com (L.B.); pietro.buzzini@unipg.it (P.B.); 4 Department of Food Biotechnology and Microbiology, Institute of Food Sciences, Warsaw University of Life Sciences—SGGW, Nowoursynowska 159C, 02-776 Warsaw, Poland; anna_kot@sggw.edu.pl

**Keywords:** yeast, carotenoids, pigments

## Abstract

Carotenoids are an essential group of compounds that may be obtained by microbiological synthesis. They are instrumental in various areas of industry, medicine, agriculture, and ecology. The increase of carotenoids’ demand at the global market is now essential. At the moment, the production of natural carotenoids is more expensive than obtaining their synthetic forms, but several new approaches/directions on how to decrease this difference were developed during the last decades. This review briefly describes the information accumulated until now about the beneficial effects of carotenoids on human health protection, their possible application in the treatments of various diseases, and their use in the food and feed industry. This review also describes some issues that are linked with biotechnological production of fungal and yeasts carotenoids, as well as new approaches/directions to make their biotechnological production more efficient.

## 1. Introduction

**Figure 1:**
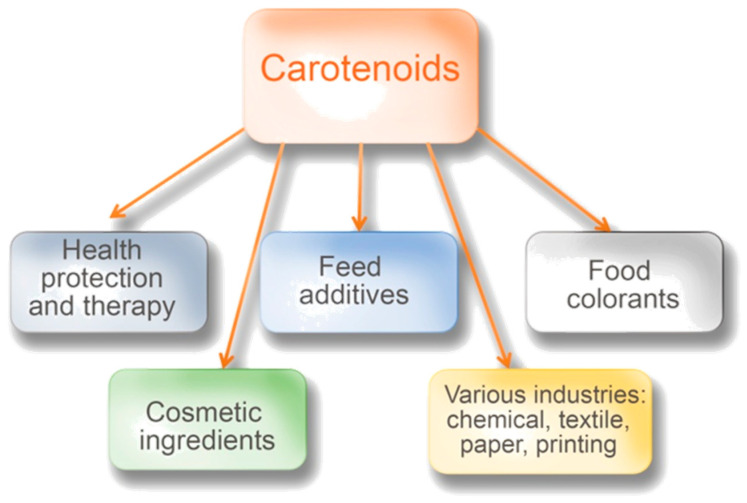
Current and potential use of carotenoids.

Carotenoids are an essential group of compounds that can be synthesized by some bacteria, yeasts, and molds. They are largely produced by plants, especially green leafy plants, for which some of them play a crucial role in photosynthesis [1,2,3,4]. In this process, they help absorb light but also play an important role in removing excess solar energy [5]. In the case of microorganisms, the main role of carotenoids is to protect cells against the negative influence of reactive forms of oxygen and radiation [6]. Carotenoids have applications in various areas of industry, medicine, agriculture, and ecology. A lot of information has been accumulated during the last decades about their possible health-protecting effects [7,8,9,10,11]. It is known that carotenoids cannot be synthesized in humans and animals. Therefore, people and animals need to obtain them from their diet [4,12]. Carotenoids may provide cosmetic benefits [7,10,13]. Moreover, their great importance in food production, as natural colorants, is well-known (Figure 1). The global market of carotenoids grew very promptly: In 2017, it reached the value of 1.5 billion USD. Based on the expectations of experts, it should reach $2.0 billion by 2022, at a compound annual growth rate of 5.7% for the period of 2017–2022 [14].

Carotenoids are lipid-soluble, mainly terpenoid pigments of 40 carbon atoms. It is thought that the most important structural characteristic of carotenoids is their conjugated double bonds (CDBs) that are largely responsible for their physicochemical properties. For instance, CDBs are responsible for the color of most carotenoids. At least seven CDBs are necessary for obtaining a colored carotenoid [10]. Carotenoids can be divided into two groups. One of them is “oxygen-free carotenes”, e.g., α-carotene, β-carotene, ¥-carotene, lycopene, and torulene. The second group is “oxygen-containing xanthophylls”, e.g., astaxanthin, lutein, zeaxanthin, β-cryptoxanthin, fucoxanthin, and canthaxanthin [4,15]. Carotenoids can also be divided into provitamin A and non-provitamin A compounds [16]. The major provitamin A carotenoids are β-carotene, α-carotene, and β-cryptoxanthin. The carotenoids that are mainly studied so far are β-carotene, lycopene, astaxanthin, lutein, and zeaxanthin [17].

It is well-known that filamentous fungi and yeasts may produce, besides carotenoids, a lot of other various pigments, including melanins, flavins, phenazines, quinones, and others. One rather new pigment being researched is the red pigment accumulated by *Saccharomyces cerevisiae* mutants for *ADE1* and *ADE2*, the product of the polymerization of 1-(5′-phosphoribosyl)-5-aminoimidazole containing several amino acid residues. This red pigment is a mixture of polymers containing a different number of monomers (4–10) and is characterized by a molecular weight from 2 to 10 kDa [17,18,19,20].

## 2. Characteristics of Some Fungal Carotenoids

The group of yeast that can synthesize carotenoids includes *Phaffia rhodozyma* (and its teleomorph *Xanthophyllomyces dendrorhous*) and species of the genera *Rhodosporidium, Rhodotorula, Sporobolomyces,* and *Sporidiobolus* [21,22,23]. Among the molds, the *Blakeslea trispora* species is of the greatest importance [24]. The biosynthesis of carotenoids in fungal cells begins with the conversion of acetyl-CoA, which is formed in the process of β-oxidation of fatty acids in the mitochondria. According to the pathway of mevalonic acid, several biochemical reactions catalyzed by specific reductases, kinases, and decarboxylases produce a five-carbon carotenoid precursor, isopentenyl pyrophosphate (IPP). The addition reactions of three IPPs lead to the formation of geranyl–geranyl pyrophosphate (GGPP), with 20 carbon atoms per molecule. The condensation of the two GGPP particles, catalyzed by phytoene synthase, produces phytoene (C40). It is a precursor to lycopene biosynthesis. Depending on the type of microorganisms, lycopene can be next transformed into β-carotene, γ-carotene, torulene, lutein, torularhodin, zeaxanthin, and astaxanthin [25].

### 2.1. β-Carotene

**Figure 2:**
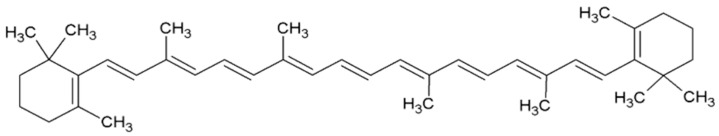
The structural formula of β-carotene.

β-carotene is an isoprenoid compound with the chemical formula C_40_H_56_ and a molecular weight of 536.88 g/mol (Figure 2). The molecule of this compound consists of two β-ionone rings connected by a polyene chain containing nine conjugated double bonds. Due to the structure of β-carotene and the system of double bonds, this compound shows a maximum absorbance at 450 nm and is characterized by a color from yellow to orange [26,27]. One molecule of this compound can be converted by specific intestinal enzymes into two molecules of vitamin A, and therefore β-carotene is the main source of this vitamin in the diet [26]. On an industrial scale, it is obtained by chemical and biotechnological methods, using the microalgae *Dunaliella salina* or *Bl. trispora* mold [28]. Efficient producers of microbial β-carotene also include the yeast *Rhodotorula glutinis* [6,29], *Rhodotorula mucilaginosa* [30], and *Sporidiobolus pararoseus* [31,32].

### 2.2. Astaxanthin

**Figure 3:**
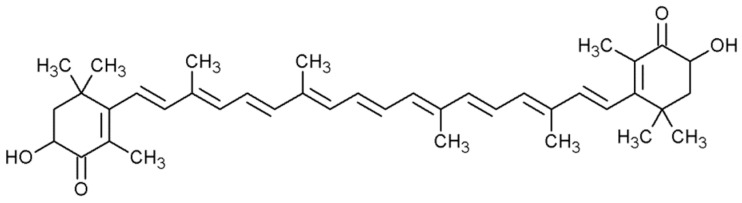
The structural formula of astaxanthin.

Astaxanthin (C_40_H_52_O_4_, 596.85 g/mol) (Figure 3) belongs to the group of xanthophylls. Two polar β-ionone rings are connected by a non-polar chain. Each ring contains one hydroxyl group and one ketone group. In total, there are 13 double bonds in the astaxanthin molecule, which determines the strong antioxidant properties of this compound. The presence of ketone and hydroxyl groups gives astaxanthin the ability to esterify and determines its polar character. Due to the presence of hydroxyl groups in the β-ionone rings, astaxanthin is an optically active compound. Chiral centers occur at positions C-3 and C-3′, and, therefore, there are three isomers of astaxanthin: enantiomers (3S, 3′S and 3R, 3′R) and a meso form (3R, 3′S) [33,34,35]. The main fungal producer of this compound is the *Xanthophyllomyces dendrorhous* yeast, which synthesizes mainly the (3R, 3′R) isomer [36].

### 2.3. Torulene

**Figure 4:**
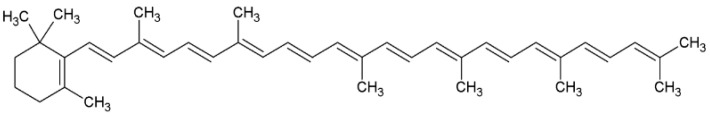
The structural formula of torulene.

Torulene (C_40_H_54_, 534.9 g/mol) belongs to the carotenes group. The torulene molecule is composed of one β-ionone ring with a polyene chain containing 12 conjugated double bonds (Figure 4). It is orange or orange-red in color, which depends on the concentration. The main microbial producers of torulene are yeasts belonging to the genus *Rhodotorula* [6], the yeast species *Sporidiobolus pararoseus* [37,38], and molds of the genus *Neurospora* [39]. Torulene has antioxidant [40] and anticancer properties [41].

### 2.4. Torularhodin

**Figure 5:**
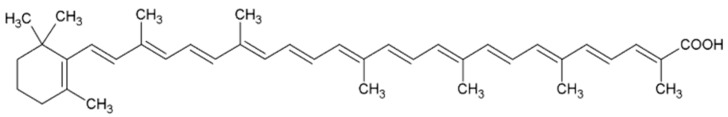
The structural formula of torularhodin.

Torularhodin (C_40_H_52_O_2_, 564.84 g/mol) has a structure similar to torulene. The only difference is the presence of a carboxyl group at the end of the polyene chain (Figure 5). For this reason, this compound belongs to the group of xanthophylls. Torularhodin shows a polar character and dark pink color [6]. The main microbial producers of this compound are *Rh. mucilaginosa* [42,43] and *Sporobolomyces ruberrimus* yeast [44,45].

## 3. Carotenoids and Human Health

It is well-known that carotenoids are compounds that are very important for human health. They can prevent a deficiency in vitamin A, which is known as the essential compound for the promotion of growth, embryonal development, and visual function. The lipophilicity of carotenoids determines their subcellular distribution; they are enriched in membranes and other lipophilic compartments, i.e., lipid droplets [16]. It is supposed that carotenoids in membranes can protect them as antioxidants. Besides that, polar carotenoids can regulate membrane fluidity [21,22]. One of their functions is linked to the protection of our vision. The deficiency of carotenoids can lead to blindness, and as it was reviewed in the literature it is a serious problem for children, especially in developing countries [23,46]. Carotenoids are vital for the protection of the retina by preventing cataracts and age-related macular degeneration [46,47,48]. There is definite evidence that shows the efficiency for eye health of lutein and zeaxanthin. They may reduce the risk for age-related macular eye diseases and lead to the improvement of visual performance that also includes positive effects, such as contrast sensitivity, glare tolerance, and photo-stress recovery [49].

Torularhodin is a carotenoid produced mainly by the yeast genera *Rhodotorula* and *Sporobolomyces*; it has strong antimicrobial properties and may become a new natural antibiotic [50,51,52,53]. The antimicrobial properties of torularhodin can also be used in the production of films for coating medical implants [54,55].

The efficiency of carotenoids’ use is known for the protection and therapy of various chronic diseases. chronic diseases. They exhibit an anti-inflammatory property and may activate the immune response of an organism [56]. It was shown that the use of lycopene-enriched foods might decrease the risk of developing atherosclerosis and other cardiovascular diseases [57,58,59]. Such beneficial results are most likely linked to the ability of lycopene to reduce systemic and high-density lipoprotein-associated inflammation and to modulate high-density lipoprotein functionality [60]. It was shown that supplementation with lycopene significantly decreased systolic blood pressures [59,61]. Astaxanthin has also been reported to exert a preventive action against atherosclerotic cardiovascular diseases by the reduction of oxidative stress and inflammation and the enhancement of lipid metabolism and glucose metabolism [62]. Supplying the body with astaxanthin allows us to effectively reduce the negative effects resulting from the oxidation and degradation of cellular elements. Another study revealed that lycopene might limit the release of proinflammatory cytokines and chemokines [63]. One study also speculated that lycopene might affect the immune functions modulating the cellular redox environment and cell-to-cell interactions and influence anti-inflammatory transcription factors, such as peroxisome-proliferator-activated receptor [64]. Several results, summarized by Rao and Rao [59], reported the involvement of lycopene and β-carotene in bone health and in preventing or decreasing the risk of osteoporosis. Such a positive effect of lycopene in decreasing osteoporosis risk was also shown in postmenopausal women [65]. Similar effects were also described for β-cryptoxanthin [49]. Lycopene consumption was demonstrated to improve bone strength, by reducing bone resorption, and to protect from type 2 diabetes, by enhancing glucose homeostasis [66,67,68].

The role of various carotenoids in the prevention of other chronic diseases was also studied [59]. Moreover, the use of lycopene in the cases of male infertility led to the improvements of sperm motility, sperm motility index, sperm morphology, and functional sperm concentration, and finally resulted in a 36% increase of successful pregnancies [59]. The possible use of lycopene in recovering the cases of alcohol-induced liver injury was also suggested [21]. Carotenoids might have beneficial effects on weight management and obesity [49,69]. It is expected that future studies could reveal a positive role of carotenoids in the treatments of other diseases, i.e., skin disorders, rheumatoid arthritis, periodontal diseases, and others [49,70].

β-Carotene and lutein have positive effects on cognitive performance [49]. The putative positive role of lycopene in the treatments of neurodegenerative diseases, including Alzheimer’s disease, was also studied [60,71]. It was thought that lutein is linked to the possible control of inflammation-related neurodegenerative disorders [72], while torularhodin can be used as a neuroprotective agent against H_2_O_2_-induced oxidative stress, due to its strong antioxidant activity [73]. Lycopene exhibited protection against amyotrophic lateral sclerosis disorder in humans [59,74].

Interesting information was received, in recent years, regarding the possible medical application of a red pigment that accumulated in *S. cerevisiae* mutants. It is known that “conformational diseases” in humans and animals are linked to abnormal aggregation of proteins and the formation of amyloid fibrils. The red pigment accumulated in *S. cerevisiae* mutants for *ADE1* and *ADE2* can bind amyloid fibrils and disturb their interactions with chaperones that, in turn, lead to the inhibition of prion “multiplication” and amyloid fibril formation [75,76,77]. It was also shown that yeast mutants, which accumulate this pigment, had lower amyloid content than wild-type parental strains. It was shown that this red pigment accumulation reduced cloned human amyloid-β aggregation. The conclusion was made that red yeast pigment has potential importance in therapy for Alzheimer’s and Parkinson’s diseases [19,20].

Carotenoids have characteristics of antioxidants [78,79,80,81]. They quench ^1^O_2_ and increase the levels of glutathione and glutathione peroxidase [4,82,83]. β-Carotene can be used for sun protection and sunburn prevention [16,84]. Carotenoids are efficient blue-light filters; they protect against photo-oxidative damages lipids, proteins, and DNA, thus preventing premature ageing of the skin and skin cancer [16,84,85,86]. It was also suggested that astaxanthin might be used as a potential anti-ageing agent [87]. β-Carotene reduces the risk of developing neoplastic diseases, and also inhibits the promotion and progression of neoplasms.

Very promising findings were also obtained on the putative efficiency of using carotenoids against some types of cancer [46,88]. The anticancer activity of some carotenoids, i.e., α-carotene, β-carotene, lycopene, torulene, torularhodin, and some others, was studied regarding prostate, breast, colon, lung, oral, gastric, and skin cancers, in addition to hepatoma, leukemia, uveal melanoma, etc. [4,52,53,66,88,89,90,91,92,93,94,95,96]. Synergistic inhibition of prostate and breast cancer cell growth was evident under the influence of combinations of low concentrations of various carotenoids [97]. The use of reporter gene assays of the transcriptional activity of the androgen receptor in hormone-dependent prostate cancer cells and the electrophile/antioxidant response element (EpRE/ARE) transcription system enabled the observation of combinations of several carotenoids (e.g., lycopene, phytoene, and phytofluene) to synergistically inhibit the androgen receptor activity and activate the EpRE/ARE system and suggested their use in the therapy and prevention of this type of cancer [97]. In the experiments performed by Prakash et al. [98], estrogen-receptor (ER) positive MCF-7 and ER-negative Hs578T and MDA-MB-231 human breast cancer cells were treated with carotenoids. Among them, β-carotene significantly reduced the growth of MCF-7 and Hs578T cells, and lycopene inhibited the growth of MCF-7 and MDA-MB-231 cells. Similar effects were also shown for astaxanthin [62]. Authors concluded that carotenoids inhibit the growth of both studied breast cancer cell lines, indicating that estrogen receptor status is an important factor for the responsiveness of breast cancer cells to carotenoid treatments [98]. The use of food rich in various carotenoids was found to decrease the risk of lung and stomach cancers [81,99,100]; although, in the case of lung cancer, negative results were obtained for β-carotene for smokers and asbestos workers. In these cases, β-carotene supplementation was associated with an increased risk of lung and gastric cancers [49,101]. It is supposed that the cancer-preventive effects exhibited by various carotenoids might also be linked to their induction and stimulation of intercellular communications via gap junctions, which are important for the regulation of cell growth, differentiation, and apoptosis [21]. More recently, lycopene was found to inhibit tumor metastasis by slowing down cell-cycle progression and inhibiting the proliferation of diverse cancer cell lines [66]. A detailed description of the different effects and mechanisms of anticancer activity of carotenoids (cell-cycle arrest, apoptosis-inducing effect, and anti-metastasis effect) is reported in some recent reviews [4,102,103].

Carotenoids may act as chemoprotective agents against cellular mutagenesis and malignant transformation [79,81,104,105,106]. Protective effects expressed by β-carotene and other carotenoids were demonstrated against the mutagenic potential of 8-methoxypsoralen, cyclophosphamide, 1-methyl-3-nitro-1-nitrosoguanidine, benzo(α)pyrene, quinolones, and ultraviolet light, using *Salmonella typhimurium* as a cell model system [81,107,108,109,110]. β-Carotene and other carotenoids (canthaxanthin, α-carotene, and lycopene) can inhibit malignant transformation induced by 3-methylcholanthrene, or X-ray treatment in the fibroblast cell line [81,111,112].

**Figure 6:**
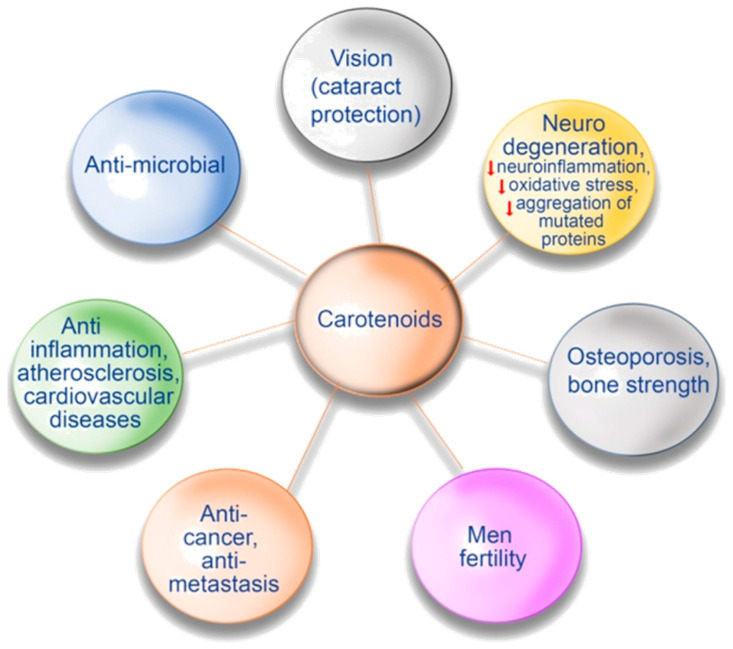
Possible positive effects of carotenoids on human health.

Antioxidant and anti-ageing effects of astaxanthin led to its wide use in cosmetics [113]. Besides all of these examples of the positive effects of carotenoids on human health, there are also data that β-carotene and astaxanthin may have immunoprotective effects, whereas lutein can prevent oxidative stress in eye tissues, as well as has antiviral activity against hepatitis B virus [113]. Figure 6 briefly presents the possible positive effects of carotenoids for human health described above.

## 4. Carotenoids and Other Microbial Pigments as Feed Additives and Colorants

Carotenoids are widely used in salmon and trout farming and in the poultry and food industry as feed additives and natural food colorants, which can give from yellow to red colors [7,114,115,116]. From an economic viewpoint, astaxanthin is the third most important carotenoid after β-carotene and lutein, due to its importance in aquaculture, and the chemical, pharmaceutical, and food industries [3]. In salmon and trout farming, it is widely used as a pigment for fish meat. Feed supplementation of carotenoids essentially improves the health of poultry birds and enhances the quality of eggs and meat. Carotenoids are very important for the pigmentation of egg yolk, skin, legs, beak, comb, feather, and fat. The use of carotenoids as alternative feed ingredients gives the possibility to replace synthetic medicine and nutrients in poultry industry [117].

Many yeasts belonging to different genera have been extensively studied during the last decades as potential efficient producers of various pigments (especially of mixtures of carotenoids). Among them, the most forthcoming is *Ph. rhodozyma* [118,119]. The production of astaxanthin has been scaled-up to the industrial level in the last decades [116,120,121]. On the other hand, the yellow carotenoid pigment zeaxanthin can be used as an additive in poultry, as well as in the cosmetics and food industries. Canthaxanthin is another carotenoid pigment that is already used in aquafeed for farmed salmonids [122]. Besides these food-related applications of carotenoids, they may serve as alternative coloring agents that are in demand in different industries, such as the textile, plastic, paint, paper, and printing industries [122,123,124,125].

The analysis of food consumer requirements revealed a growing rejection of synthetic food dyes during the last decade. The use of some synthetic colorants in food and cosmetic processing has recently been banned due to their hyperallerginicity, carcinogenicity, and other toxicological problems [122,123]. For example, the astaxanthin produced chemically is not approved for human consumption, due to the presence of by-products [126]. Correspondingly, a growing demand for dyes of natural origin is becoming increasingly more popular. It is well-known that natural coloring agents can be extracted from various plants, algae, and microorganisms (i.e., bacteria, yeasts, and fungi), which can produce various pigments [127]. Some food-grade microbial pigments are already produced biotechnologically. Among them is the hydroxyanthraquinoid pigment Arpink red, which is produced by a strain of *Penicillium oxalicum* var. *armeniaca* isolated from soil by the Czech company Ascolor Biotech s.r.o. The patent covering Arpink Red also claims its anticancer effects for applications in the food and pharmaceutical fields [127]. Another example is the yellow vitamin riboflavin (vitamin B_2_). It can be produced by the yeast species *Meyerozyma* (formerly *Candida*) *guilliermondii* or *Debaryomyces subglobosus* and by the dimorphic fungus *Eremothecium ashbyi* (and its heterotypic synonym *Ashbya gossypii*); the latter is used for industrial-scale production [126,128].

Recent studies explored the possibility of replacing the use of yellow pigments from the fungus *Monascus* sp. (which are not approved for the use in EU and USA, because of the risk of possible contamination by the nephrotoxic and hepatotoxic metabolite citrinin), with similar pigments produced by non-mycotoxigenic strains of the fungal genus *Talaromyces* [124,128,129,130,131,132]. Marine fungi are also studied as promising sources of novel pigments [128]. New findings in this area that give new possibilities for modern biotechnology are described in detail in the review by Dufosse et al. [128].

## 5. Biotechnology

Some carotenoids are now produced at the industrial level, using microbial strains. Although the fungal species *Mucor circinelloides, Phycomyces blakesleeanus*, and *Bl. trispora* are well-known producers of β-carotene, *Bl. trispora* is the main one used for industrial production. This species is very interesting in biotechnology; however, the need to co-cultivate (+) and (–) sexual mating types of this fungus makes this technology quite complex. This species is nonpathogenic and nontoxigenic [127,133]. The first biotechnological company that started the production of β-carotene in Western Europe, at the industrial level, between 1995 and 2001, was the Dutch company Gist-brocades (now DSM); meanwhile, in the Soviet Union, production of β-carotene in Eastern Europe started a decade earlier [131]. The Spanish Company Vitatene (now DSM) started the production of lycopene from *Bl. trispora* for the European market in 2003 [131].

One of the approaches for further improvement of these technologies may be connected with the use of new selected or mutant strains, as well as the improvement of fermentation conditions [126,134,135]. Carotenoids can be synthesized by different groups of microorganisms. However, given the efficiency of the biosynthesis process and economic factors, yeasts of the genus *Rhodotorula* deserve special attention. Another possible approach to increase the production of carotenoids may include applying different stress factors. It was shown that, in the case of oxidative, osmotic, and salt stress, *Rh. glutinis*, *Rh. mucilaginosa,* and *Sporidiobolus* (formerly *Sporobolomyces*) *salmonicolor* produce significantly higher amounts of carotenoids [116,136,137]. In a recent study, it was revealed that low temperature caused an increase in the biosynthesis of carotenoids by *Rhodotorula toruloides* (formerly *Rhodotorula gracilis*) in media containing agro-industrial waste-potato wastewater and glycerol. The induction of osmotic stress and low temperature intensified the biosynthesis of β-carotene (up to 73.9% of the total carotenoid content). In the conditions of oxidative stress, the yeast synthesized torulene (up to 82.2%) more efficiently than under other conditions, whereas white-light irradiation increased the production of torularhodin (up to 20.0%) [138].

One more efficient approach to this problem was the engineering of the carotenoid pathway [46,139]. New achievements reached in genetic and metabolic engineering of microorganisms made it possible to optimize host microorganisms to use as advanced microbial cell factories. It was demonstrated that the best combinations of mutations identified for β-carotene production were also beneficial for the production of lycopene [140]. It was shown that recombinant microbial cell factories can be engineered on the basis of an oleaginous yeast, *Yarrowia lipolytica*, to produce astaxanthin by submerged fermentation [126]. Recently, a study directed to metabolic engineering of *S. cerevisiae* demonstrated the potential of a yeast-based process for β-carotene production [141]. Recent reviews give insights into microbial engineering principles for the overproduction of carotenoids and describe key strategies and current advances in engineering of the metabolism of carotenoid-producing microorganisms for maximizing carotenoid production [140,142,143]. It was reported that chemical mutagenesis led to the obtaining of *Bl. trispora* strains, which produced 100-fold higher amounts of β-carotene, compared with the wild-type strain [134]. The same organism is also proposed for the industrial production of lycopene [134,144]. It is expected that further identification of genes important in the carotenogenic pathway will be reached in the next few years and will lead to obtaining higher quantities of carotenoids at the industrial level [116].

Because of the economic efficiency, biotechnological production of carotenoids can be significantly increased when the costs are diminished by the use of waste or by-products from other biotechnologies as the main substrate for microorganisms. Various ideas are proposed accordingly [126,134,135,145]. In this context, corn syrup, sugarcane bagasse, wheat bran, rice bran, silage, whey, and crude glycerin are alternatives for producing carotenoids [146,147]. These products are found in abundance due to the production of biodiesel, sugar, and corn processing [148,149,150,151]. Interesting research showed that, besides the raw glycerin from biodiesel production, spent brewer’s yeast from the breweries may also be used to substitute carbon and nutrient sources, to produce carotenoids (β-carotene, torularhodin, torulene, and γ-carotene) by *Rhodotorula* strains [152]. An interesting proposal for carotenoids production using spent coffee grounds was published. Considering that, at the moment, coffee is the second largest product in the world (after petroleum) and that the industrial production of instant coffee in 2012 yielded about 330.000 tons of spent coffee grounds, this idea seems rather promising. The best results on carotenoids’ production using spent coffee grounds was obtained by using the yeast species *Sporobolomyces roseus* (other strains they have studied were *Rh. glutinis, Rh. mucilaginosa*, and *Cystofilobasidium capitatum*) [153,154]. Three yeast strains isolated in the Brazilian forests, belonging to the species *Sporidiobolus pararoseus*, *Rh. mucilaginosa*, and *Pichia fermentans*, were found to produce cryptoxanthin and β-carotene when they were cultivated in the media containing parboiled rice water and crude glycerol or parboiled rice water and sugar cane molasses [155,156].

There are also some other possibilities of using industrial wastes. For example, researchers have explored the possibility of obtaining simultaneously high-value carotenoids and lipids for biodiesel production [157]. The yield of synthesized carotenoids (mainly, β-carotene) using *Rh. glutinis* cultivated on brewery wastewater as a carbon source was rather low, but the idea is interesting and needs further investigations and development [158]. Utilization of agro-industrial waste in fermentation is an important source that may provide nutrient sources for the fast growth of microorganisms and enhances their pigment production. Utilization of these substrates also reduces the accumulation of biomass in large quantities, which may cause deterioration to the environment [122,159]. Fruit wastes derived from orange, pomegranate, and pineapple can be used as a culture medium for β-carotene production from *Rh. mucilaginosa* (formerly *Rh. rubra*) [160]. Working in the same direction, a set of cold-adapted pigmented yeast strains were isolated from plants and food samples. Some yeast strains that may synthesize both carotenoids and extracellular enzymes—lipases and cellulases—were identified (*S. roseus* and *S. pararoseus*). Because of this, it is expected that these strains might be interesting for the development of biotechnological production of carotenoids using cheap substrates, such as agro-industrial waste, including also lignocellulose [160]. Some studies showed the possibility to develop a new bioprocess that gives possibility to produce β-carotene from the xylose fraction of lignocellulosic biomass, using engineered *S. cerevisiae* strain [161,162].

Thinking of the large-scale biotechnological production of various carotenoids, it is clear that various strategies must be used. Besides the already mentioned above selection of over-producing strains, obtaining genetically modified hyper-producing strains and metabolic engineering of strain-producers, and the use of cheap carbon and nitrogen sources from waste and by-products from other existing technologies, it is necessary to remember that carotenoid synthesis in microorganisms depends on the number of factors, which are summarized in the review by Mata-Gomez et al. [3]. These factors are carbon and nitrogen sources, light, temperature, aeration, metal ions, and especially some trace elements, and the addition of some chemicals (such as ethanol and acetic acid) into the growth medium. It was shown that the use of carbon sources, such as ethanol, could provoke an increase in the synthesis of pigments [3,120,163,164]. Their production is positively affected by white light [3,165]. Temperature is another important factor which influences carotenoids synthesis [166]. It was revealed for *Rh. glutinis* that the temperature of 25 °C favored the synthesis of β-carotene and torulene, while a temperature range of 30–35 °C favored the torularhodin biosynthesis [167,168].

Correct aeration is one more important factor affecting carotenoid biosynthesis [149]. An increased rate of stirring led to an essential increase in carotenoid production by *Rh. mucilaginosa* [169]. The addition of 0.5 M NaCl to the fresh water used in the preparation of nutrient media for engineered carotenoids producing *S. cerevisiae* strain increased the production of β-carotene by almost two times. An increase in the C:N ratio further improved carotenoid production by this strain [170]. The nitrogen sources were revealed as the main factors that most efficiently influenced the intracellular accumulation of carotenoids in yeast *Rh*. *mucilaginosa* [169,171]. Metal ions (such as Ba, Fe, Mg, Ca, Zn, and Co) and especially some trace elements (such as Al, Zn, and Mn) are very important for carotenoids synthesis in various species of *Rhodotorula* [170]. A recent study has described the potential of the ascomycetous yeast species *Y. lipolytica* as a β-carotene-producing cell factory, reporting the highest titer of recombinant β-carotene produced to date [172]. The medium optimization (C:N ratio, possible addition of glycerol) led to an improvement of up to 50% in the yield of β-carotene production in the best of the conditions [173,174]. Some other approaches were also proposed to increase pigment production. One of them is the immobilization of culture-producers. Alipour et al. found that the addition of the natural loofa sponge immobilized *Rh. mucilaginosa* (formerly *Rh. rubra*) in a cell-immobilized airlift photobioreactor considerably increased the production of carotenoids [175].

At the same time, in summarizing the existent literature, it was concluded that, despite the large amount of the information linked with various issues of carotenoids’ effects, their importance, and the biotechnology of their production published during last years, some of these substances that have health-promoting activities still require further study. These are lutein, zeaxanthin, α-carotene, β-cryptoxanthin, phytoene, torulene, and torularhodin [10].

## 6. Conclusions

Carotenoids represent a very large group of various compounds. These pigments have been studied for more than 120 years [176]. During this time, a substantial amount of information regarding their chemistry, structure, and mechanisms of functional activities has been accumulated. Due to the health-promoting properties of carotenoids, there is a growing interest in the methods of obtaining and enriching them, primarily in food products. For many years, they have been widely used in salmon and trout farming and the poultry and food industry, as feed additives and natural food colorants. The increase in the demand for carotenoids in the global market was essential during the last years, and it is expected that there will be an annual growth rate of their production at the level of 5.7%. The main quantity of these compounds produced by industry has synthetic origin. At the same time, during the last decades, there has been a marked increase in negative associations of these pigment consumers with the use of synthetic carotenoids, some of which may be hyperallergenic, carcinogenic, and may have some other toxicological problems. The alternative is to use natural carotenoids from plants or those produced by microorganisms. Currently, the production of natural carotenoids is more expensive than obtaining their synthetic forms, but a lot of new approaches/directions on how to decrease this difference was recently developed. The first strategy is based on the use of industrial waste and by-products; the further search for new efficient strain-producers, as well as the selection of existing industrial strains; and the application of physiological, metabolic, and genetic-engineering methods. The second strategy is to obtain carotenoids from invasive plant species that cause enormous economic and infrastructural damage and loss of biodiversity. Examples of such plants are Japanese knotweed (*Fallopia japonica* Houtt.) and Bohemian knotweed (*Fallopia* x *bohemica*) [24]. It is well-known that, besides carotenoids, microorganisms synthesize many other pigments that are also of essential importance for various areas of human activities and are actively studied or already produced in large amounts by modern biotechnology. Some of them were briefly mentioned in this review, and plenty of special reviews devoted to them were recently published [125,177,178]. However, the information about the potential application of carotenoids that were obtained during the last decades testifies that they are still underestimated. We expect that the unique characteristics of carotenoids will lead to further demand for natural carotenoids in the coming years and much more efficient use of these compounds in various areas, including those associated with the prevention and therapy of various human diseases. Simultaneously, this should give an additional incentive to further study this unique group of natural pigments.
